# Real-Time Anomaly Detection in Physiological Parameters: A Multi-Squad Monitoring and Communication Architecture

**DOI:** 10.3390/s25030929

**Published:** 2025-02-04

**Authors:** Razaq Jinad, Khushi Gupta, Damilola Oladimeji, Amar Rasheed, Cihan Varol

**Affiliations:** Department of Computer Science, Sam Houston State University, Huntsville, TX 77341, USA; raj032@shsu.edu (R.J.); kxg095@shsu.edu (K.G.); dko011@shsu.edu (D.O.); cxv007@shsu.edu (C.V.)

**Keywords:** anomaly detection, physiological parameters, military, health monitoring

## Abstract

In military operations, real-time monitoring of soldiers’ health is essential for ensuring mission success and safeguarding personnel, yet such systems face challenges related to accuracy, security, and resource efficiency. This research addresses the critical need for secure, real-time monitoring of soldier vitals in the field, where operational security and performance are paramount. The paper focuses on implementing a machine-learning-based system capable of predicting the health states of soldiers using vitals such as heart rate (HR), respiratory rate (RESP), pulse, and oxygen saturation SpO_2_. A comprehensive pipeline was developed, including data preprocessing, the addition of noise, and model evaluation, to identify the best-performing machine learning algorithm. The system was tested through simulations to ensure real-time inference on real-life data, with reliable and accurate predictions demonstrated in dynamic environments. The gradient boosting model was selected due to its high accuracy, robustness to noise, and ability to handle complex feature interactions efficiently. Additionally, a lightweight cryptographic security system with a 16-byte key was integrated to protect sensitive health and location data during transmission. The results validate the feasibility of deploying such a system in resource-constrained field conditions while maintaining data confidentiality and operational security.

## 1. Introduction

Military personnel often face some of the most challenging and demanding duties, which are compounded by various psychological and physiological stressors [1]. Physiologically, military personnel often carry out various operations in harsh and challenging locations, which can involve extreme temperatures, such as high altitudes [2], humid jungles [3], or arid deserts. In addition to these environmental challenges, military personnel endure rigorous physical demands, involving prolonged periods of strenuous activity [4], sleep deprivation [5], irregular meal schedules, and inadequate nutrition. These factors can significantly impact military performance in combat and lead to reduced alertness and cognitive function [6].

Psychological stressors include prolonged periods of separation from family and loved ones, exposure to life-threatening situations, combat-related trauma, and the constant pressure to perform at peak levels in high-stakes environments. The psychological toll of these stressors can manifest in various ways, such as heightened anxiety, post-traumatic stress disorder (PTSD), depression, and other mental health challenges. The cumulative effect of these stressors can further exacerbate the challenges faced by military personnel. For instance, high levels of stress and anxiety can impact sleep quality and recovery, leading to fatigue, decreased cognitive function, and impaired decision-making abilities.

Given these challenges, robust monitoring of vital signs such as heart rate, blood oxygen level, and breathing rate [7,8] is essential to ensure that soldiers remain fit and capable of performing their duties effectively, even in the most challenging environments [9,10]. Furthermore, the Tactical Combat Casualty Care (TCCC) guidelines have also recommended vital sign monitoring [11]. To support this, the Internet of Battlefield Things (IoBT) has become essential. The interconnection and collaborative decision-making between combat equipment and battlefield resources is one of the characteristics of the Internet of Battlefield Things (IoBT) [12].

The concept of IoBT was proposed by the U.S. Army Research Laboratory (ARL) to enable predictive analytics for intelligent command and control services [13]. As an important component of the IoBT, soldiers are the most flexible information nodes on the battlefield. Heterogeneous sensors integrated into a soldier’s equipment provide a command center with multidimensional battlefield information. Meanwhile, as an interdependent and interconnected group of entities, soldiers constantly communicate, coordinate, and jointly plan and execute tasks using the equipment of the IoBT.

According to the US Army Research Institute of Environmental Medicine [7], the concept of a soldier physiological status monitor (PSM) must extend from operational performance monitoring to tactical casualty alerts, triage, and medical management capabilities. The concept should incorporate the following requirements:

- Provide continuous, real-time physiological health and performance status information to soldiers, unit leaders, commanders, and battalion medical staff using an intra-soldier network.
- Key sensor measurements that offer actionable soldier performance insights, such as performance capabilities, accurate predictions, trend detection, and recommend interventions through the use of AI (artificial intelligence).
- Lightweight wireless monitoring capability that can be employed by a squad leader and that ensures security through low-power encryption mechanisms, improving medical situational awareness.

Traditional wired communication networks are not only costly but also susceptible to errors caused by factors such as distance, battlefield topography, and weather conditions. In contrast, modern wireless communication systems offer easy installation, scalability, and the capability to connect soldiers seamlessly regardless of ecological challenges [14]. A well-functioning wireless communication network is the key to achieving success in combat operations [15,16].

Establishing secure real-time communication links between the troops and the command and control center is a critical component of combat operations [17]. In addition to advanced safety measures, having secure communication facilities for soldiers is increasingly essential for modern combat operations, facilitating communication, navigation, health monitoring, video links, and more. To ensure the success of a mission and the safety of the soldiers, real-time situational information must be shared [18]. A soldier can be equipped with an IoT combat equipment that monitors his/her vital parameters. This information can then be shared with with other soldiers and communicated with the command and control center [19,20] to inform treatment and prioritization decisions.

Building upon established PSM requirements, in this research, we propose and simulate a multi-squad physiological monitoring and communication architecture designed for the battlefield. This framework focuses on the continuous assessment of vital physiological parameters, such as heart rate, pulse, breathing rate, and blood oxygen levels, at an individual soldier level. By leveraging advanced sensor technology, we collect real-time data on these critical metrics, ensuring that any deviations from the normal values are identified in a timely manner. Central to our architecture is a local machine-learning model that analyzes the collected data to determine whether the readings fall within acceptable thresholds. In instances where anomalies are detected, the system notifies both the base station and fellow squad members via secure communication channels. This facilitates immediate intervention, thereby enhancing the overall safety and operational readiness of soldiers in high-stakes environments. Our proposed architecture not only strengthens situational awareness on the battlefield but also promotes a proactive approach to soldier health emergencies.

The rest of this study proceeds in the following manner: Section II discusses the current literature, while Section III details our simulation architecture. Section IV describes the proposed system architecture, Section V explains our implementation and results, and lastly, Section VI concludes the study.

## 2. Literature Review

In this section, we mention some of the previous works carried out in physiological monitoring and secure communication frameworks among soldiers. This will help us identify the gaps in the literature which we aim to fill with our research.

Khan et al. [21] presented a solution for enhancing soldier safety and operational efficiency. It integrates wearable sensors, GPS, and GSM modules to continuously monitor soldiers’ vital signs, such as heart rate and body temperature, while simultaneously tracking their location. The system transmits real-time data to a central base station, allowing commanders to monitor health parameters and coordinate troop movements effectively. In emergencies, soldiers can use an onboard alert button to send their location and trigger immediate responses. The system is built with components like Arduino microcontrollers, LM35 temperature sensors, LDRs, and GSM communication, offering a portable design.

Building on this idea, Gondalia et al. [16] outlined an IoT-driven healthcare system for war soldiers, combining body sensor networks (BSNs), GPS, ZigBee, and LoRaWAN technologies. The system tracks soldier health parameters like heart rate, temperature, and humidity, and monitors their position in real-time. ZigBee facilitates communication among soldiers, while LoRaWAN enables long-range data transmission from squad leaders to the control unit, overcoming cellular network limitations in high-altitude or remote war zones. The collected data are analyzed on a cloud platform using the K-means clustering algorithm, which predicts conditions like health status or potential hazards based on sensor input, aiding decision-making during battlefield operations. By leveraging machine learning and real-time analytics, the system offers insights into soldiers’ health and environmental risks, enhancing strategic planning and emergency response.

Furthermore, Bandopadhaya et al. [22] proposed an integrated healthcare monitoring solution for soldiers using IoT technology and distributed computing. It features a three-layer service-oriented architecture comprising a wireless sensor body area network (WSBAN) layer, a fog layer, and a cloud layer. At the WSBAN layer, sensors collect real-time healthcare parameters like body temperature, pulse rate, and oxygen levels. A Mamdani fuzzy inference engine is used to classify the data into “well”, “alarming”, and “critical” categories, with only the alarming data being transmitted to the fog layer, thereby reducing the computational burden. In the fog layer, time-series pattern analysis is performed using polynomial regression to identify and classify health status patterns. Then, a Sugeno fuzzy model categorizes these patterns into “momentary instable” (MI), “abnormal”, and “reinvestigate” classes. Only the data classified as “abnormal” are transmitted to the cloud layer for comprehensive analysis and visualization.

Bin et al. [23] proposed a framework for secure communication and the mental health assessment of soldiers in the battlefield. The framework integrates secure communication, real-time health monitoring, and situational awareness using wearable sensors, including heart rate, respiration, and neural activity monitors. By employing the Advanced Encryption Standard (AES) in counter mode, it ensures secure and efficient communication suitable for resource-constrained devices. The system facilitates robust communication through ZigBee and IEEE 802.11ah technologies, for both intra-node and inter-node interactions. Additionally, it supports continuous health monitoring, with a focus on mental stress evaluation, enhancing battlefield decision-making, and reducing mortality rates. Mental stress is assessed by analyzing physiological and neural signals processed with machine learning algorithms, achieving an accuracy of 83.33%.

Moreover, Raza et al. [24] introduced a lightweight system designed to enhance soldier safety and operational effectiveness in warfare. The system integrates biomedical sensors, GPS, GSM, and microcontrollers to monitor soldiers’ health parameters, including heart rate and body temperature, and transmits the data to a base station. It also enables soldiers to send coded messages in emergencies. The base station processes and displays the data in real-time using a GUI, with alerts indicating critical conditions like injuries or death. A correlation formula combining heart rate and body temperature was proposed to accurately predict health status and validated through experimental testing on diverse human conditions. The system ensures continuous monitoring and updates through SMS and visual feedback, offering a reliable means to support strategic decisions during missions.

Integrating safety into a health monitoring system, Kenag et al. [25] presented a robust framework that integrates advanced sensors for capturing health data such as heart rate, body temperature, and respiration rate, alongside biometrics like iris patterns for authentication. The framework also introduces a multilayer inference system (MIS) to optimize the use of wireless body area networks (WBAN) and low-power wide-area networks (LPWAN) for the real-time health monitoring and location tracking of soldiers. The MIS significantly reduces battery consumption by minimizing the frequency of sensor data transmissions. Key features of the framework include real-time physiological monitoring, emergency alarm notifications triggered by health or activity changes, and multifactor authentication using biometric data combined with health information for secure identification of soldiers and equipment.

Finally, Shi et al. [26] explored the development of wearable military devices through a multi-level fusion framework (MLFF) to enhance battlefield situational awareness and soldier safety. With the rise of the Internet of Battlefield Things (IoBT), soldiers, as key nodes, interact with heterogeneous sensor networks integrated into body sensor networks (BSNs). The MLFF combines raw data, high-level analyses, and decision-making processes to improve the resilience and functionality of soldier BSNs. It monitors diverse parameters like behavior, physiology, fatigue, emotions, environmental factors, and location under battlefield constraints. The proposed framework emphasizes multi-source data fusion to enhance real-time decision-making, reduce false alarms, and improve soldier safety and mission success rates.

The existing literature presents significant advancements in soldier health monitoring and communication systems, yet gaps remain in their integration and real-time responsiveness in dynamic battlefield environments. While solutions like those by Khan et al. [21] and Raza et al. [24] focus on continuous health monitoring and positional tracking, they are limited by reliance on GSM networks and lack robust anomaly detection mechanisms. Approaches such as Gondalia et al.’s [16] use of machine learning and cloud platforms address network limitations but introduce latency and computational dependency on centralized systems, which may not be viable in real-time critical scenarios. Systems like Bandopadhaya et al.’s [22] three-layer architecture and Bin et al.’s [23] secure communication frameworks improve data management and security but lack an emphasis on intra-squad communication and immediate intervention capabilities. Our research bridges these gaps by introducing a decentralized, multi-squad physiological monitoring and communication architecture that leverages local machine learning models for real-time anomaly detection, secure squad-level communication, and immediate response. This ensures enhanced situational awareness and proactive intervention, addressing the critical need for both individual and collective soldier safety in high-stakes environments.

## 3. Simulation Architecture

**Figure 1:**
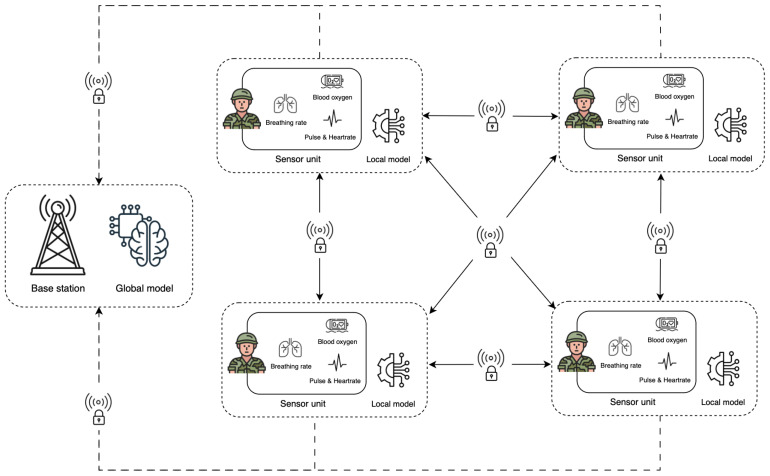
Simulation architecture of the framework on a battlefield.

In this section, we outline the key components of the simulation architecture for the proposed framework, as illustrated in Figure 1. It illustrates a multi-layered system that facilitates an individual and squad-level health monitoring framework that leverages sensor units, local machine learning models, and a base station monitoring unit for continuous analysis.

- **Internet of Battlefield Things (IoBT):** This architecture aligns with the IoBT concept, where interconnected devices on the battlefield collect and share data to enhance situational awareness. Here, soldiers are acting as flexible information nodes, providing multidimensional battlefield information to the command center and fellow squad members.
- **Sensor Units on Soldiers:** Each soldier is equipped with a sensor unit that monitors key physiological parameters, including heart rate, breathing rate, and blood oxygen levels. These metrics are captured locally and analyzed by a local machine learning model. This local model assesses if the values fall within safe, normal ranges or if there are anomalies that might indicate unusual health parameters.
- **Local Analysis with Machine Learning:** The local model in each soldier’s sensor unit performs real-time analysis of vital signs. If a reading deviates significantly from the expected thresholds (indicating, for example, an elevated heart rate or low blood oxygen level), the model flags this as an anomaly.
- **Communication between Soldiers and the Base Station:** If an anomaly is detected, the system sends an alert through a secure communication channel. This alert is transmitted to other squad members and the base station, which serves as the command center for monitoring and decision-making. This allows squad members and command to respond immediately, enabling timely intervention in case of potential emergencies.
- **Base Station and Global Model:** The base station houses a global model that aggregates data from multiple sensor units across the squad(s) and provides a comprehensive view of the health status of all monitored soldiers. These aggregated data can be used for further predictive analytics, helping to support decision-making at the command level and allowing for proactive measures in critical situations.
- **Interconnected Communication Flow:** The secure wireless channels (indicated by locks on the communication lines) illustrate the intra-squad and squad-to-base station communication pathways. These pathways are crucial for maintaining the real-time awareness of each soldier’s physiological state and ensuring that alerts can be swiftly communicated across all necessary entities within the battlefield network.

## 4. Proposed System Architecture

**Figure 2:**
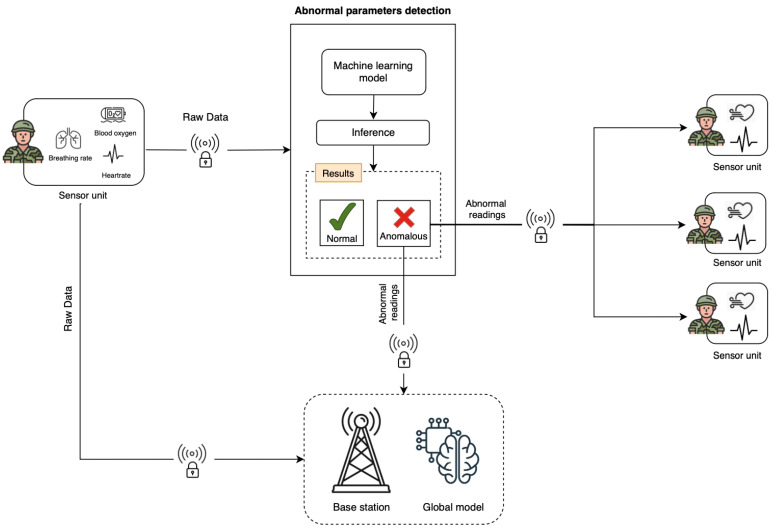
The proposed system architecture.

This research proposes an anomaly detection architecture for real-time physiological monitoring and communication within military squads. Figure 2 provides a magnified view of the anomaly detection architecture within each local node. Each soldier is equipped with a sensor unit that continuously monitors vital physiological parameters such as breathing rate, blood oxygen levels, pulse, and heart rate. The data collected are referred to as raw data, which are processed locally at each soldier’s node. This initial processing is handled by an integrated local machine-learning model that evaluates the data to check for deviations from predefined normal thresholds. If the data falls within acceptable ranges, the system classifies it as normal, filtering out non-critical information to reduce unnecessary transmissions. However, if deviations are detected, the data are flagged as anomalous, which triggers a secure communication alert. This alert is sent both to nearby squad members and to a base station for immediate intervention and situational awareness. The localized alerts ensure that fellow soldiers in the squad are promptly informed, facilitating rapid on-site responses to potential health emergencies.

Simultaneously, the abnormal readings are transmitted to the base station, which plays a central role in aggregating and analyzing the data at a higher level. The base station hosts a global machine learning model capable of synthesizing data from multiple soldiers to provide comprehensive situational awareness. This global model supports predictive analytics by identifying trends, patterns, and potential health risks across the squad. Additionally, the base station serves as a feedback loop by updating the local machine learning models in the soldiers’ sensor units based on new patterns or insights, ensuring continuous system improvement and adaptability.

This architecture achieves two critical objectives: immediate anomaly detection at the soldier level to trigger rapid responses, and centralized oversight at the base station for strategic decision-making. By combining localized anomaly detection with centralized situational analysis, the system ensures a scalable and flexible solution for real-time health monitoring. This approach enhances soldier safety, facilitates proactive intervention, and supports operational efficiency in dynamic, high-stakes battlefield environments.

### 4.1. Dataset

The dataset used in this research was sourced from the Beth Israel Deaconess Medical Center (BIDMC) [27], a publicly available dataset commonly utilized for analyzing physiological parameters in clinical research. The data were collected from a cohort of patients at the Beth Israel Deaconess Medical Center and are widely used in the research community for studying vital signs and health monitoring. We chose this dataset due to its high-quality, continuous recordings of essential physiological parameters, making it ideal for developing machine learning models aimed at patient state classification.

The dataset contains time-series measurements of key physiological parameters. Each of these parameters plays a crucial role in assessing the health status of individuals, as they are commonly monitored in clinical settings to detect abnormalities or changes in a patient’s condition. The dataset includes these parameters recorded continuously over an 8-min duration, allowing for a detailed analysis of their variation under different conditions. The total number of aggregated rows for all patients was 25,493.

Data collection was performed across a sample of 53 subjects, with each subject monitored over varying periods. The key parameters of the dataset are heart rate (HR), oxygen saturation (SpO_2_), respiratory rate (RESP), and pulse rate.

- HR: The heart rate measured in beats per minute (bpm).
- SpO_2_: The oxygen saturation level in percentage (%), reflecting the efficiency of oxygen transport in the blood.
- RESP: The respiratory rate, measured in breaths per minute, which is an indicator of breathing function.
- Pulse Rate: The pulse rate in beats per minute (bpm), which often correlates closely with heart rate but is measured via a different sensor.

The focus of this research was on these four parameters (heart rate, SpO_2_, respiratory rate, and pulse rate), rather than the raw physiological signals. By analyzing these parameters, the goal was to classify the physiological state of individuals under various simulated conditions, such as changes in altitude, temperature, and physical activity levels.

### 4.2. Feature Extraction and Engineering

**Figure 3:**
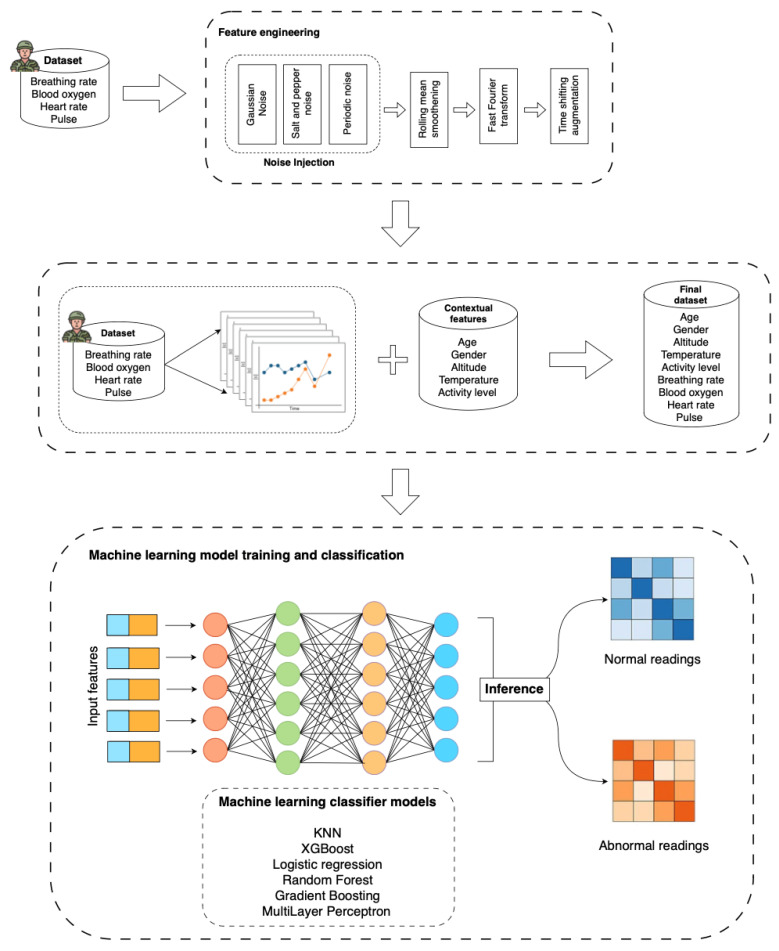
Machine learning pipeline.

The feature engineering process plays a crucial role in extracting meaningful attributes from raw physiological data, allowing for more accurate classification of individuals’ states. The dataset used in this research included time-series data of heart rate (HR), pulse, respiratory rate (RESP), and oxygen saturation (SpO_2_). To enhance the robustness of the data and ensure that the machine learning models could generalize well in real-world scenarios, a series of preprocessing steps and feature extraction techniques were applied. Figure 3 shows a pipeline describing the feature engineering.

Initially, noise was introduced into the dataset to simulate the variability typically observed in real-life measurements. This included adding Gaussian noise to mimic random fluctuations from sensor inaccuracies and salt-and-pepper noise to replicate occasional spikes or dropouts in the signals. Furthermore, periodic noise was introduced into the respiratory rate signal to model regular fluctuations that might arise due to environmental factors or physiological rhythms. To smooth out these noisy signals, a rolling mean was applied, which reduced short-term variations, while preserving long-term trends in the data.

In addition to these dynamic adjustments, frequency-domain analysis was applied to extract features from the physiological signals. The fast Fourier transform (FFT) was utilized to capture the dominant frequencies within each signal, which are indicative of underlying physiological patterns. For instance, frequency components in the heart rate signal can provide insights into heart rate variability, which is often associated with stress or autonomic regulation.

To further enhance the dataset, time-shifting augmentation was employed to generate additional training samples. This technique involved shifting the time-series data forward or backward to simulate slight variations in the data collection process, thereby increasing the robustness of the models to temporal misalignment. Finally, contextual features related to environmental factors (such as altitude and temperature) and personal factors (such as age and gender) were integrated into the dataset to capture the influence of external conditions on physiological parameters.

The labeling process for classifying physiological states as either normal or abnormal was designed to dynamically adjust based on contextual factors (altitude, temperature, and activity level) and personal characteristics (age and gender). We established personalized thresholds for heart rate, respiratory rate, SpO_2_, and pulse, taking into account these factors to create more realistic and individualized ranges. For example, higher altitudes lead to lower baseline SpO_2_ levels, while intense physical activity naturally elevates heart rate and respiratory rate. We then compared each individual’s measured vital signs against these dynamically adjusted thresholds. If any of the measurements fell outside the computed normal range, the individual was labeled as being in an abnormal state; otherwise, they were labeled as normal. This adaptive labeling approach ensured that the classification was context-sensitive, accounting for variations that might be expected under different environmental conditions or for individuals of different ages and genders.

After the comprehensive feature engineering process, our final dataset consisted of the following features:

Noisy Signals: These features represent the original physiological signals with added Gaussian noise to simulate real-world measurement variability. This helps the models generalize better to noisy data.

- HR_noisy: Heart rate with added noise.
- SpO_2__noisy: Oxygen saturation with added noise.
- RESP_noisy: Respiratory rate with added noise.
- Pulse_noisy: Pulse rate with added noise.

Smoothed Signals: These features are derived by applying a rolling mean to the noisy signals to reduce short-term fluctuations and highlight longer-term trends

- HR_smooth: Smoothed heart rate.
- SpO_2__smooth: Smoothed oxygen saturation.
- RESP_smooth: Smoothed respiratory rate.
- Pulse_smooth: Smoothed pulse rate.

Frequency-Domain Features: These features are obtained using fast Fourier transform (FFT) to extract the dominant frequency components from the physiological signals. Frequency analysis provides insights into the periodicity and variability of the signals

- HR_fft: Dominant frequency component of the heart rate.
- SpO_2__fft: Dominant frequency component of the oxygen saturation.
- RESP_fft: Dominant frequency component of the respiratory rate.
- Pulse_fft: Dominant frequency component of the pulse rate.

Contextual and Demographic Factors: These features capture the personal and environmental context under which the measurements were taken. These factors were integrated to account for how physiological parameters vary based on external conditions:

- Age: Age of the individual.
- Gender: Gender of the individual.
- Altitude: Altitude level at the time of measurement.
- Temperature: Ambient temperature during measurement.
- Activity_level: Intensity of physical activity during measurement.

Target Variable: The final feature is the target label used for classification. The target label is denoted as state and indicates whether the individual’s physiological state is normal or abnormal.

Overall, these features provide a rich dataset that captures both the physiological signals and the contextual factors that influence them, enabling the machine learning models to more accurately classify the physiological state of individuals in both controlled and real-life simulation settings.

### 4.3. An AI-Based Anomaly Detection Engine for Physiological Parameter Exchange in the Battlefield

After the feature engineering process, the preprocessed dataset was used to train a range of machine learning models to classify the physiological state of individuals. The classification task was framed as a binary classification problem, where the models aimed to predict whether a given set of physiological parameters indicated a normal or abnormal state. The first model implemented was a random forest classifier, which leverages an ensemble of decision trees to make robust predictions. This model was chosen due to its ability to handle high-dimensional data and its resistance to overfitting. In parallel, a logistic regression model was also trained to serve as a baseline, due to its simplicity and interpretability. To explore the effectiveness of distance-based algorithms, a K-nearest neighbors (KNN) classifier was included, which uses the proximity of data points to make predictions.

To capture more complex, nonlinear patterns in the data, a gradient-boosting classifier and an XGBoost classifier were also employed. These models are known for their high performance in classification tasks, due to their ability to combine weak learners into strong predictive models through boosting techniques. Additionally, a multi-layer perceptron (MLP), a type of feedforward artificial neural network, was utilized to explore the potential of deep learning for this task. The MLP was configured with multiple hidden layers to capture deeper representations of the input features.

Overall, the combination of advanced feature engineering techniques and a diverse set of machine learning models allowed for a thorough exploration of the dataset, leading to a more accurate classification of physiological states under varying conditions. The integration of environmental and personal factors, along with the simulation of real-world noise, helped in creating a robust system capable of detecting abnormal states in real-time scenarios.

## 5. Implementation and Results

This section describes the setup and results of the two experiments conducted for our simulation study. The first experiment focused on developing and evaluating a machine learning pipeline to detect the physiological state of individuals based on their vital signs, while the second experiment simulated real-time monitoring of soldiers in the field to detect their state dynamically.

### 5.1. Experimental Setup

For the experimental setup, we utilized Google Colab Pro as a computational platform to conduct the machine learning experiments for detecting physiological states. The computational resources provided by Google Colab Pro included access to a Tesla T4 GPU, which is particularly efficient for handling computationally intensive tasks. The system was equipped with 12.7 GB of RAM, 10 GB of GPU memory, and disk space of 107.7 GB, ensuring ample resources for effectively training and evaluating the models. The entire experiment was implemented using the Python programming language, leveraging key libraries such as sci-kit-learn, PyEMD, Scipy, NumPy, and Pandas. The development environment for this phase was Jupyter Notebook, which facilitated interactive coding and experimentation.

We utilized a different hardware setup for the soldier simulation experiments to simulate real-time field conditions. These simulations were conducted on a MacBook Pro, equipped with 8 GB of RAM and 512 GB of storage. The simulation was implemented in Python using Visual Studio Code (VS Code) as the integrated development environment (IDE). This configuration provided a lightweight yet capable setup for testing real-time detection of soldiers’ physiological states, allowing for efficient deployment and monitoring of the models developed in the initial experiment phase.

### 5.2. Results

**Figure 4:**
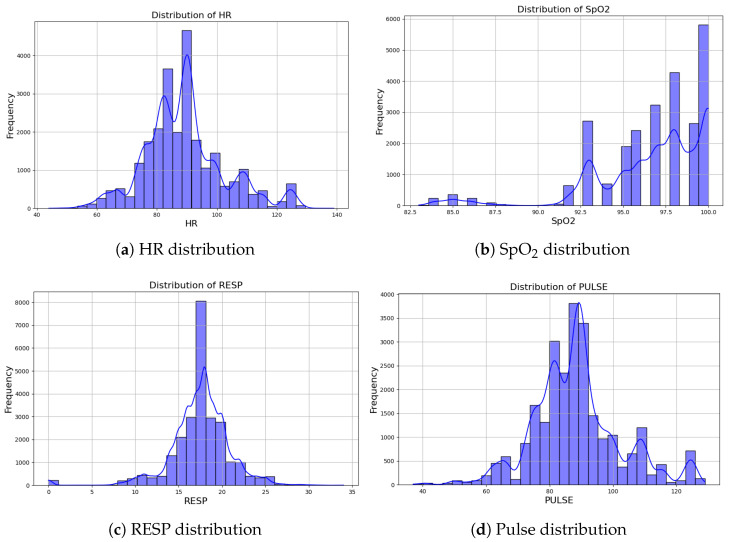
Distribution of signals.

**Figure 5:**
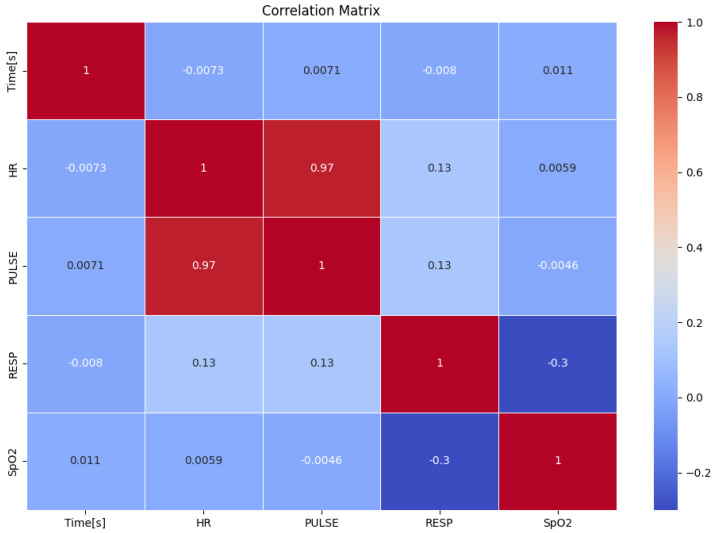
Correlation of original features.

This section contains the visualizations and results for the machine learning model pipeline and the soldier simulation experiments. First, we performed some exploratory analysis on the initial dataset to better understand the nature of the data. We visualized the data to obtain insights into the data. Figure 4 and Figure 5 show the distribution and correlation of the features, respectively.

The distribution plots for the vital features reveal significant insights into the dataset. The heart rate (HR) shows a bimodal trend with peaks around 80 and 100 bpm, predominantly ranging between 60 and 120 bpm, which aligns with typical healthy ranges, though some extreme values suggest potential anomalies. The SpO_2_ (oxygen saturation) distribution is heavily concentrated near 100%, consistent with normal physiological levels, but with a few lower values. The respiratory rate (RESP) distribution displays a sharp peak of around 18 breaths per minute, which corresponds to the average adult breathing rate, with most values falling between 10 and 25 breaths per minute. The pulse distribution closely mirrors that of the heart rate, with peaks near 80 and 100 bpm, suggesting a strong correlation between these two features. These patterns highlight the predominantly normal physiological states represented in the dataset, while also hinting at outliers or abnormalities that could be critical for state detection.

The correlation matrix illustrates the relationships among the features in the dataset. A strong positive correlation of 0.97 is observed between heart rate (HR) and pulse, confirming their physiological dependence. The respiratory rate (RESP) shows weak positive correlations with both HR and pulse (approximately 0.13), indicating minimal direct relationships with these features. SpO_2_ displays a mild negative correlation with RESP (−0.3), which might reflect physiological phenomena like oxygen desaturation during respiratory abnormalities, but it shows negligible correlation with HR and pulse. The time feature appears to have no significant correlation with any of the other variables. Overall, the matrix highlights that HR and pulse are strongly related, while the other features are largely independent, suggesting complementary roles in predicting soldier states.

#### ML Pipeline Results

For the model evaluation, the dataset was split into training and testing subsets. The models were evaluated on the test set using standard performance metrics such as accuracy, precision, recall, and F1-score. To ensure robustness, cross-validation was performed using a stratified k-fold approach. This method helped to mitigate potential biases by ensuring that the training and validation sets had a similar distribution to the target classes. Table 1 shows the performance of the models.

The logistic regression model achieved an accuracy of 85%, with comparable precision and recall values. While it performed adequately, its F1-score of 0.80 suggests a slight imbalance in its ability to effectively handle true positives and false positives. This makes it suitable as a baseline model but less effective for capturing complex patterns in data. The k-nearest neighbors (KNN) classifier showed a notable improvement, achieving an accuracy of 89%, along with consistent precision, recall, and F1-score values of 0.89. This indicates that the distance-based algorithm was able to leverage the feature space effectively, though it may still struggle with high-dimensional or overlapping data distributions.

The random forest classifier demonstrated excellent performance, with a balanced accuracy, precision, recall, and F1-score of 0.92. This suggests that its ensemble approach was highly effective in capturing the relationships between features, making it a reliable choice for this classification task. Similarly, gradient boosting achieved an accuracy of 92%, with a slightly higher precision (0.93) and F1-score (0.93) compared to the random forest. This indicates its superior ability to handle imbalanced classes and complex feature interactions through iterative boosting techniques.

The XGBoost classifier closely followed with an accuracy of 91%, showing robust and consistent metrics. While slightly lower than gradient boosting in overall performance, XGBoost’s computational efficiency and flexibility make it a strong contender in practical applications. Lastly, the multi-layer perceptron (MLP) also achieved an accuracy of 92%, with balanced precision, recall, and F1-score metrics. This demonstrates its ability to learn nonlinear patterns in the data, making it comparable to ensemble models like random forest and gradient boosting in terms of effectiveness.

**Figure 6:**
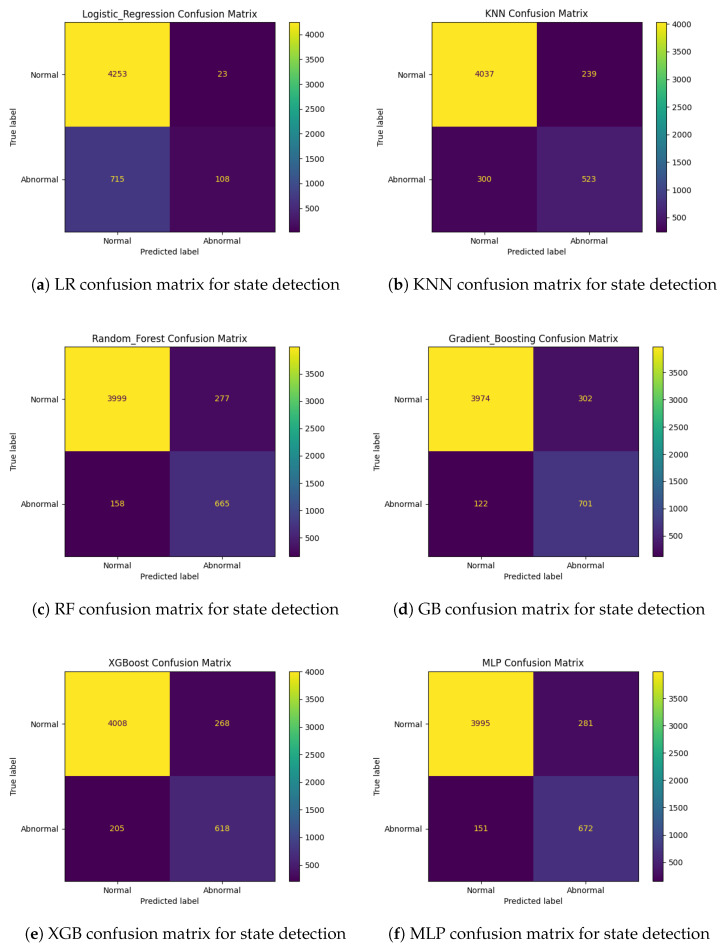
Confusion matrices for ML models.

The results for each model were further analyzed by generating confusion matrices, as visualized in Figure 6. These visualizations were used to understand the models’ performance, distinguishing between normal and abnormal states. The confusion matrices provided insights into the types of errors made by each model, helping to identify areas where the model performance could be improved.

Overall, the results suggest that the ensemble methods (random forest and gradient boosting) and neural networks (MLP) were the most effective models for this pipeline. They offer both high accuracy and balanced metric performance, making them well-suited for real-world applications where robust detection of abnormal states is critical.

### 5.3. Simulation

**Figure 7:**
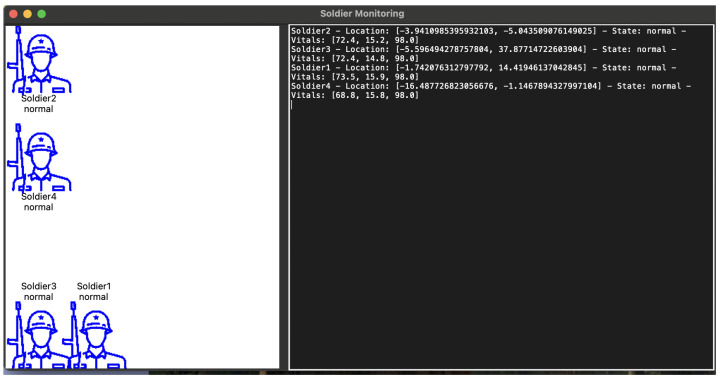
Simulation showing soldiers with normal states.

**Figure 8:**
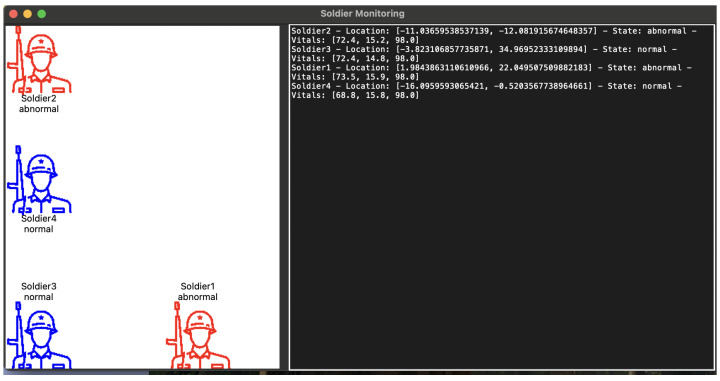
Simulation showing some soldiers in abnormal states.

In the experimental simulation, we developed a comprehensive soldier monitoring system that implemented a distributed network of soldiers communicating with a central base station. Figure 7 and Figure 8 depict the experiment. The simulation experiment was designed to evaluate the real-time performance of the deployed machine learning model in a simulated field environment. The system architecture consisted of multiple interconnected components, including individual soldier nodes, a base station for centralized monitoring, and a graphical user interface for real-time visualization and interaction. Based on the prior evaluation, the gradient boosting classifier was selected as the best-performing model for deployment, due to its high accuracy, robustness to noise, and efficient handling of complex feature interactions.

This classifier is inherently well-suited to managing variability in physiological parameters across diverse environmental conditions, due to its ability to handle non-linear relationships and interactions between features effectively. The algorithm’s iterative nature enables it to learn complex patterns and adapt to noisy and imbalanced data. This is crucial for tasks involving dynamic physiological signals influenced by environmental and personal factors. In our experiments, the gradient-boosting classifier demonstrated robustness when faced with simulated noise and variability in vitals, including contextual factors such as altitude, temperature, age, and activity levels. During the simulation, the model demonstrated reliable inference on real-life data, ensuring accurate predictions of soldier states in dynamic and resource-constrained environments. The goal of the simulation was to replicate real-life conditions, continuously monitor vital signs, and classify the physiological state of simulated soldiers in real time.

The simulation emulated the collection of real-time physiological data from multiple soldiers in the field. Each soldier’s data stream was represented by a synthetic dataset, continuously generated with randomized variations to reflect real-world conditions. Gaussian, periodic, and salt-and-pepper noise were introduced to simulate sensor inaccuracies and environmental fluctuations. The data streams were preprocessed in real time using the same feature engineering pipeline used during model training. The simulation ran on a multi-threaded architecture, with each soldier operating on their own thread to ensure smooth, concurrent operation. The machine learning model processed the vital signs data to classify each soldier’s state as either normal or abnormal, demonstrating the potential for automated health monitoring and early warning systems in military applications. The update frequency was set to 5 s for vital sign generation and state updates, providing a balance between real-time monitoring and system resource utilization.

While the current model focuses on short-term classifications of physiological parameters, it still provides reliable and actionable insights into critical states such as fatigue or abnormal physiological responses. By incorporating features like dynamic threshold adjustments based on environmental conditions (e.g., altitude, temperature, and activity level) and individual factors (e.g., age and gender), the system can effectively identify when a soldier is approaching a fatigued state or experiencing abnormal physiological changes, even within shorter timeframes. For example, prolonged elevated heart rate, reduced oxygen saturation, or irregular respiratory patterns are detected by the system as signs of stress or exhaustion.

Additionally, the system’s ability to continuously classify physiological states in real-time ensures that it can monitor transitions in a soldier’s condition during operations. While the model does not explicitly track long-term trends using sequential data analysis methods, it can still infer when a soldier becomes fatigued based on cumulative short-term abnormal classifications. This functionality provides timely alerts that are critical for decision-making during missions.

During the simulation, soldiers’ movements were tracked across a normalized coordinate system (0–100 for both x and y axes), and their vital signs were continuously monitored and shared. The system demonstrated robust performance in maintaining consistent communication between all nodes, while providing real-time updates to the graphical interface. The addition of the soldier status monitoring feature proved particularly valuable, as it allowed for immediate access to detailed information about any soldier’s perception of their teammates’ status, enhancing the overall situational awareness capabilities of the system.

We also implemented a cryptographic security system using the Advanced Encryption Standard (AES), with a 16-byte key to ensure encrypted communication and safeguard sensitive health and location data during transmission. The AES was selected for its robust balance of strong encryption and high performance, making it particularly suitable for resource-constrained environments, such as real-time operations on devices with limited computational power, while maintaining robust security.

Furthermore, regarding real-time processing latency, the AES demonstrated minimal impact on the system’s overall performance. The encryption and decryption processes were lightweight enough to operate seamlessly alongside the machine learning pipeline. Additionally, the implementation ensured that cryptographic operations occurred in parallel with data preprocessing and inference tasks, further mitigating any latency issues.

This encryption mechanism is critical for real-world applications, where unauthorized access to soldier positions or health data could compromise operational security. Communication between soldier nodes and the base station is secured using TCP/IP protocols, ensuring reliable data transmission, even over long distances or in environments with blocked signals. The system includes automatic reconnection mechanisms and data buffering to handle the temporary communication interruptions common in field operations, while each soldier node maintains a local cache of recent vital signs and status information to ensure continuous operation during brief network outages.

Moreover, to minimize latency, data packets are compressed before transmission, and preprocessing is performed locally on each soldier’s device using lightweight machine learning models. Only critical alerts or anomalies are transmitted to the base station, reducing the communication overhead. A scalable multi-threaded architecture enables each soldier node to operate independently, allowing concurrent data processing without bottlenecks, while adaptive resource allocation effectively handles high-concurrency scenarios.

## 6. Conclusions

In this research, we aimed to develop and evaluate a system for measuring and predicting the vital states of soldiers in the field using machine learning models. The primary objectives included collecting and analyzing vital signs such as heart rate (HR), pulse, respiratory rate (RESP), and oxygen saturation (SpO_2_); performing exploratory data analysis (EDA); selecting the best machine learning model; and simulating real-time deployment. We conducted a series of experiments, including statistical analysis, distribution plotting, and correlation analysis, to understand the relationships between features and assess their predictive importance. Various models were tested and evaluated based on their accuracy and efficiency, with the best-performing model recommended for deployment in the simulation. Assumptions such as data consistency, minimal noise in sensor readings, and computational resource availability in the field were made to ensure reliable implementation. The system, when deployed, offers real-time insights into soldiers’ health states, enhancing situational awareness and decision-making capabilities, which could significantly improve outcomes in mission-critical environments.

In future work, we will focus on improving the system’s robustness and scalability for real-world deployment. This includes incorporating environmental factors (e.g., temperature, altitude, and humidity) and behavioral metrics to enhance accuracy; optimizing resource efficiency for edge devices; and exploring ensemble learning, sensor fusion, and advanced deep learning models. Adversarial training methods, including generating adversarial examples during training, will be investigated to improve resilience against attacks. Additionally, implementing error detection and correction mechanisms, such as cyclic redundancy checks (CRC), will help safeguard data integrity. Extensive field testing and validation will ensure adaptability to diverse operational conditions and refine the system performance.

## Figures and Tables

**Table 1:** Metric comparison of ML models for state detection.

Models	Accuracy	Precision	Recall	f1-Score
Logistic Regression	0.85	0.85	0.85	0.80
k-Nearest Neighbors	0.89	0.89	0.89	0.89
Random Forest	0.92	0.92	0.92	0.92
Gradient Boosting	0.92	0.93	0.92	0.93
XGBoost	0.91	0.91	0.91	0.91
MultiLayer Perceptron	0.92	0.92	0.92	0.92

## Data Availability

Data derived from public domain resources.
